# In Older Men, Lower Plasma 25-Hydroxyvitamin D Is Associated with Reduced Incidence of Prostate, but Not Colorectal or Lung Cancer

**DOI:** 10.1371/journal.pone.0099954

**Published:** 2014-06-20

**Authors:** Yuen Y. E. Wong, Zoë Hyde, Kieran A. McCaul, Bu B. Yeap, Jonathan Golledge, Graeme J. Hankey, Leon Flicker

**Affiliations:** 1 Western Australian Centre for Health and Ageing, Centre for Medical Research, Western Australian Institute for Medical Research, Perth, Australia; 2 School of Medicine and Pharmacology, University of Western Australia, Perth, Australia; 3 Department of Endocrinology and Diabetes, Fremantle Hospital, Perth, Australia; 4 The Vascular Biology Unit, Queensland Research Centre for Peripheral Vascular Disease, School of Medicine and Dentistry, James Cook University and Department of Vascular and Endovascular Surgery, Townsville Hospital, Townsville, Queensland, Australia; 5 Department of Neurology, Sir Charles Gairdner Hospital, Nedlands, Perth, Australia; Kagoshima University Graduate School of Medical and Dental Sciences, Japan

## Abstract

**Context and objective:**

Prostate, colorectal and lung cancers are common in men. In this study, we aimed to determine whether vitamin D status is associated with the incidence of these cancers in older men.

**Design:**

Prospective cohort study.

**Setting and participants:**

4208 older men aged 70–88 years in Perth, Western Australia

**Main outcome measures:**

Plasma 25-hydroxyvitamin D [25(OH)D] concentration was measured by immunoassay. New diagnoses of prostate, colorectal and lung cancers were determined via electronic record linkage.

**Results:**

During a mean follow-up of 6.7±1.8 years, there were 315, 117 and 101 new diagnoses of prostate, colorectal and lung cancer. In multivariate competing risks proportional hazards models, every 10 nmol/l decrease in 25(OH)D concentration was associated with a 4% reduction in prostate cancer incidence (sub-hazard ratio [SHR] 0.96, 95% confidence interval [CI] 0.92–1.00). Every halving of 25(OH)D concentration was associated with a 21% reduction in incident prostate cancer in multivariate analysis (SHR 0.79, 95% CI 0.63–0.99). Following exclusion of prostate cancer cases diagnosed within 3 years of blood sampling, low 25(OH)D <50 nmol/l was associated with lower incident prostate cancer, and higher 25(OH)D >75 nmol/l was associated with higher incidence, when compared to the reference range 50–75 nmol/l, respectively (p = 0.027). Significant associations were also observed when 25(OH)D was modeled as a quantitative variable. No associations were observed between plasma 25(OH)D concentration with incidence of colorectal or lung cancer.

**Conclusion:**

Lower levels of vitamin D may reduce prostate cancer risk in older men. By contrast, levels of vitamin D did not predict incidence of colorectal or lung cancers. Further studies are needed to determine whether a causal relationship exists between vitamin D and prostate cancer in ageing men.

## Introduction

Prostate, colorectal and lung cancers are common in older males [1]. Important risk factors include increased age, smoking and physical inactivity. Previous studies have linked vitamin D deficiency to the risk of cancer, but the role of vitamin D in cancer pathogenesis is currently controversial.

Many experimental studies have documented pivotal roles of vitamin D in cancer genesis and progression. Its anti-carcinogenic qualities have been attributed to its active metabolite, 1,25-dihydroxyvitamin D3 [1,25(OH)D3], which exerts its influence via 2 pathways: the genomic and non-genomic (rapid) pathways. The genomic pathway requires the binding of 1,25(OH)D3 to the vitamin D receptor (VDR), which regulates transcription of genes involved in numerous cellular processes relevant for anti-cancer effects [2]. The non-genomic pathway involves binding of 1,25(OH)D3 to the VDR, leading to intracellular signaling, rapid activation of cellular ion channels, and subsequent protection of DNA integrity [3]. 1,25(OH)D3 also has an immune-modulatory effect which impede the development of malignancy [4]. Despite accumulating evidence from experimental studies suggesting that low vitamin D status might be a causal risk factor for cancer, a recent systematic review of prospective cohort studies have reported no association between elevated vitamin D concentrations and lower risks of most cancers, excepting colorectal cancer [5]. Similarly, prospective studies on vitamin D and cancer mortality as well as vitamin D and survival in cancer patients have yielded inconsistent findings [6]. Randomized controlled trials of vitamin D supplements have failed to show anti-cancer effects, possibly due to methodological limitations and inadequate statistical power [7]. Further observational and interventional studies are therefore warranted to clarify the potential role of vitamin D on cancer incidence.

In this study, we examined the relationship between vitamin D status and the incidence of prostate, colorectal and lung cancers in men aged 70–88 years. The primary circulating form of vitamin D, plasma 25-hydroxyvitamin D [25(OH)D] was measured as an indicator of vitamin D status. We tested the hypothesis that vitamin D level at baseline would predict the incidence of specific cancer types in a large population-based cohort of older men.

## Materials and Methods

### Study population

We conducted a prospective cohort study of participants from the Health in Men Study (HIMS), which has been described in detail elsewhere [8]. In brief, approximately 40 000 men residing in Perth, Western Australia, were randomly selected from the electoral roll. These men, aged 65–83 years, were randomized to the screening and control arms of a trial of screening for abdominal aortic aneurysm. 12 203 men participated in the screening and completed a health assessment between 1996 and 1999 (HIMS Wave 1). In 2001–2004, 5585 men responded to the second phase of this study (HIMS Wave 2) and blood samples were collected from 4249 of them. More than 95% of the participants were Caucasian. The Human Research Ethics Committee of the University of Western Australia approved the protocol for HIMS and written informed consent was obtained from the participants.

### Outcomes of interest

Cancer diagnoses and mortality information were obtained from the Western Australian Data Linkage System (WADLS), which links together data from the state cancer registry, death registry and hospital morbidity data system [9]. Notification of cancer is mandatory in Western Australia and the International Classification of Diseases for Oncology (ICDO-3) is used for cancer coding. For our analyses, we considered topography codes C33 and C34 to indicate lung cancer; C18, C19, C20 and C21 to indicate colorectal cancer; and C61.9 to indicate prostate cancer. For incident cancer cases, we included only primary invasive malignancies detected after the date of blood sampling and before December 31, 2010. Metastases, neoplasms in which primary or metastatic status was uncertain, neoplasms of unknown behavior and in situ carcinomas were all excluded.

### Explanatory variables

Using a combination of data collected at Waves 1 and 2, the following variables were available: age at Wave 2, education (completed high school or better by the end of Wave 1), living circumstance (living alone or in residential aged care facility during Wave 2), smoking status (current, former or never smoker during Wave 2), and taking calcium and vitamin D supplements during Wave 2 (yes or no). During Wave 1, the participants were asked whether they had done any vigorous exercise (apart from work) in a usual week, that would make them breathe harder or puff and pant (such as fast walking, jogging, aerobics, vigorous swimming, vigorous cycling, tennis, football, and squash). Physical activity was defined as ≥150 min of vigorous exercise in a usual week.

In order to calculate the weighted Charlson Co-morbidity Index (CCI) [10], we obtained the health records and death certificates from WADLS and evaluated the number and seriousness of the comorbid diseases. The Charlson's Index takes into account 17 common medical conditions that predict one-year mortality: myocardial infarction, congestive heart failure, peripheral arterial disease, cerebrovascular disease, dementia, chronic pulmonary disease, connective tissue disease, ulcers, liver disease, diabetes (including diabetes with end organ damage), hemiplegia, renal disease, leukaemia, lymphoma, other tumours, metastatic tumours, and AIDS [10].

Height and weight were measured in accordance with guidelines of the International Society for the Advancement of Kinanthropometry [11]. Body mass index (BMI) was calculated from height and weight in kg/m^2^.

### Biochemical analyses

Blood samples were collected during Wave 2 between 0800 and 1030. Plasma was separated from the blood samples within 1 hour of collection and stored at −80°C until assayed. As described previously [12], we measured 25(OH)D using the automated DiaSorin “LIAISON 25(OH)D TOTAL” chemilumininescent immunoassay. This was carried out on archived plasma aliquots in a series of runs performed between 2011 and 2012. The interassay coefficient of variation was 13.2% at 37.9 nmol/l and 11.3% at 131 nmol/l. The date of blood collection was documented and seasonality determined: summer (December–February), autumn (March–May), winter (June–August) and spring (September–November). Serum creatinine was measured with a Roche Hitachi 917 analyzer (Roche Diagnostics).

### Statistical analyses

Data were analysed using Stata release 11.1 (Stata Corp, College Station, TX, USA). Descriptive statistics were calculated for the demographic, lifestyle and clinical variables according to the presence or absence of each cancer of interest. Men who reported taking calcium and vitamin D supplements were excluded from all analyses. The associations between 25(OH)D and incident cancer were explored by competing risk analyses [13]. This approach was being considered due to the fact that in epidemiological studies, patients dying from non-cancer causes are usually considered as controls. These individuals might in reality be susceptible to biomarker abnormalities or to the development of cancer. The association between biomarker and cancer incidence might as a result be unrecognized due to their premature non-cancer mortality. Incident cancer was reported as sub-hazard ratio (SHR) with 95% confidence intervals (95% CI).

We defined lower vitamin D status as 25(OH)D <50 nmol/l, a threshold used widely by experts to indicate vitamin D deficiency [14]. Higher vitamin D status was defined as >75 nmol/l, a point from which parathyroid hormone levels plateau to a steady state [15]. The associations between plasma 25(OH)D concentration and cancer incidence was investigated in three different ways: according to whether 25(OH)D was <50 nmol/l or >75 nmol/l (using 50–75 nmol/l as reference), per 10-nmol/l decrease in concentration, and by halving of 25(OH)D. We transformed 25(OH)D by dividing the natural logarithm of 25(OH)D by the natural logarithm of 0.5. After this transformation, a one-unit change corresponds to a halving of the level of 25(OH)D.

To explore whether the associations between 25(OH)D and specific incident cancers were curvilinear, we entered 25(OH)D into the models as restricted cubic splines. The associations appeared curvilinear and were subsequently modeled with this approach. When 25(OH)D is modeled as categorical variables, the reported p-values test the null hypothesis that the SHRs are all equal to 1. When 25(OH)D is modeled as quantitative variables, the reported p-values test the null hypothesis that there is no linear trend between 25(OH)D and incident cancer. We performed univariate and multivariate analyses, adjusting for age, education, living circumstance, smoking status, physical activity, CCI, BMI, creatinine, seasonality and previous diagnosis of cancer (other than the cancer of interest). 397 men had a previous diagnosis of prostate cancer and were excluded from the incident prostate cancer analyses. 138 and 27 men had previous diagnoses of colorectal and lung cancer respectively, and were excluded from the incident colorectal and lung cancer analyses, respectively. To minimise the possibility of reverse causality and ascertainment bias, we repeated the univariate and multivariate analyses after excluding the incident cases diagnosed within 3 years of blood sampling. P-values<0.05 were considered statistically significant.

## Results

The demographic, lifestyle and clinical characteristics of the study population, according to the presence or absence of incident cancers of interest, are shown in Table 1. 25(OH)D was available for 4233 men. 907 men (21.4%) had 25(OH)D concentration <50 nmol/l, 1834 men (43.3%) had 25(OH)D concentration between 50 and 75 nmol/l, and 1492 men (35.3%) had 25(OH)D >75 nmol/l. Men with 25(OH)D <50 nmol/l were older in age compared to those with 25(OH)D >75 nmol/l (77.4 years vs 76.9 years, p = 0.003). The former were more likely to be current or former smokers (p = 0.005), and also had higher number of co-morbidities (p<0.001). Detailed descriptive statistics of 25(OH)D data are published elsewhere [16].

**Table 1:** Demographic, lifestyle and clinical characteristics of the study population (excluding men who took calcium and vitamin D supplements) by the end of HIMS Wave 2, according to the presence or absence of incident cancer.

	Prostate cancer			Colorectal cancer			Lung cancer		
	Yes (n = 295)	No (n = 3190)	P value	Yes (n = 102)	No (n = 3614)	P value	Yes (n = 93)	No (n = 3720)	P value
**Age, ** ***years*** a	76.8±3.6	77.0±3.6	0.394	78.5±3.7	77.0±3.6	<0.001	77.9±3.1	77.0±3.6	0.014
**Completed high school or better, ** ***n (%)***	158 (50.2)	1687 (48.0)	0.453	61 (52.1)	1910 (48.0)	0.375	44 (43.6)	1989 (48.5)	0.329
**Lived alone or in residential aged care facility, ** ***n (%)***	61 (19.4)	590 (16.8)	0.237	20 (17.1)	673 (16.9)	0.953	24 (23.8)	686 (16.7)	0.061
**Smoking, ** ***n (%)***									
**Never smoked**	115 (36.5)	1159 (32.9)	0.431	28 (13.9)	1353 (34.0)	0.009	3 (3.0)	1409 (34.3)	<0.001
**Former smoker**	184 (58.4)	2172 (61.7)		87 (74.4)	2424 (60.8)		81 (80.2)	2503 (60.9)	
**Current smoker**	16 (5.1)	190 (5.4)		2 (1.7)	208 (5.2)		17 (16.8)	195 (4.8)	
**Physical activity, ** ***n (%)***	82 (26.0)	787 (22.4)	0.135	22 (18.8)	901 (22.6)	0.331	16 (15.8)	936 (22.8)	0.099
**CCI≥5, ** ***n (%)***	4 (1.3)	130 (3.7)	0.025	3 (2.6)	140 (3.5)	0.581	3 (3.0)	167 (4.1)	0.581
**BMI, ** ***n (%)***									
**<18.5 kg/m^2^**	1 (0.3)	24 (0.7)	0.858	2 (1.7)	22 (0.6)	0.234	1 (1.0)	26 (0.6)	0.732
**18.5–24.9 kg/m^2^**	111 (35.2)	1206 (34.4)		43 (36.8)	1345 (33.9)		38 (37.6)	1379 (33.7)	
**25.0–29.9 kg/m^2^**	156 (49.5)	1771 (50.5)		52 (44.4)	2022 (50.9)		46 (45.5)	2084 (50.9)	
**≥30 kg/m^2^**	47 (14.9)	505 (14.4)		20 (17.1)	581 (14.6)		16 (15.8)	602 (14.7)	
**25(OH)D, ** ***n (%)***									
**<50 nmol/l**	55 (18.6)	704 (22.1)	0.224	27 (26.5)	789 (21.8)	0.453	24 (25.8)	808 (21.7)	0.302
**50–75 nmol/l**	124 (42.0)	1372 (43.0)		44 (43.1)	1559 (43.1)		33 (35.5)	1615 (43.4)	
**>75 nmol/l**	116 (39.3)	1114 (34.9)		31 (30.4)	1266 (35.0)		36 (38.7)	1297 (34.9)	
**25(OH)D, ** ***nmol/l*** a	70.9±22.1	68.3±23.6	0.065	67.4±24.0	68.3±23.3	0.684	69.4±24.7	68.3±23.3	0.640
**Creatinine, ** ***µmol/l*** a	91.3±21.8	94.1±32.8	0.139	96.6±42.8	93.6±31.2	0.314	94.8±26.0	93.8±31.8	0.741
**Season of blood collection, ** ***n (%)***									
**Summer/Autumn (Dec–May)**	157 (49.8)	1672 (47.5)	0.423	50 (42.7)	1911 (48.0)	0.265	51 (50.5)	1961 (47.8)	0.585
**Winter/Spring (Jun–Nov)**	158 (50.2)	1849 (52.5)		67 (57.3)	2074 (52.1)		50 (49.5)	2146 (52.3)	

Abbreviations: CCI, Charlson Comorbidity Index; BMI, body mass index; 25(OH)D, 25-hydroxyvitamin D.

a Continuous data presented as mean ± standard deviation and categorical data as n (%).

The participants were followed-up for a mean duration of 6.7±1.8 years (range 0.1–9.2 years), comprising 25 723, 27 359 and 28 089 person-years for prostate, colorectal and lung cancers, respectively. During this period, 315 men were diagnosed with prostate cancer, 117 with colorectal cancer, and 101 with lung cancer. After exclusion of all men taking calcium and vitamin D supplements as well as new cancer diagnoses occurring within 3 years of blood sampling, there were 155 men diagnosed with prostate cancer, 60 with colorectal cancer and 60 with lung cancer during follow-up. There were 943 competing risk events (death from any cause) in men included in prostate cancer analyses, 1071 in colorectal cancer analyses, and 1065 in lung cancer models.

### Association between 25(OH)D and incident prostate cancer

As illustrated in Table 2, every 10 nmol/l decrease in 25(OH)D concentration was associated with a 4% reduction in prostate cancer incidence, after adjustment for age, education, living circumstance, smoking status, physical activity, CCI, BMI, creatinine, seasonality and previous diagnosis of cancer (other than prostate) (SHR 0.96, 95% CI 0.92–1.00). Similarly, every halving of 25(OH)D concentration was associated with a 21% reduction in incident prostate cancer after adjustment for other risk factors (SHR 0.79, 95% CI 0.63–0.99). The association was weakened when 25(OH)D was modeled as categorical variables in the competing risk analyses (Table 2). To address the possibility of reverse causality, we excluded cases diagnosed within 3 years of blood sampling (Table 3). In multivariate analysis, low 25(OH)D concentration of <50 nmol/l was associated with lower incident prostate cancer (SHR 0.76, 95% CI 0.46–1.23) and higher 25(OH)D concentration of >75 nmol/l was associated with higher incidence (SHR 1.39, 95% CI 0.98–1.97), when compared to the reference range of 50–75 nmol/l, respectively (p = 0.027). Significant associations were also observed when 25(OH)D was modeled as a quantitative variable: lower 25(OH)D was associated with reduced incidence of prostate cancer in fully-adjusted analyses (per 10 nmol/l decrease: SHR 0.91, 95% CI 0.86–0.96; per halving of 25(OH)D: SHR 0.58, 95% CI 0.42–0.80).

**Table 2:** Competing risks proportional hazards models exploring associations between vitamin D and incident cancers.

	Univariate			Multivariate^a^		
	SHR	95% CI	P value	SHR	95% CI	P value
**Prostate cancer** (n = 295)						
25(OH)D (nmol/l)			0.373			0.477
<50	0.88	0.64–1.22		0.89	0.65–1.23	
50–75	1			1		
>75	1.11	0.86–1.43		1.09	0.84–1.42	
Per 10-nmol/l decrease in 25(OH)D	0.96	0.92–1.00	0.048	0.96	0.92–1.00	0.079
Halving of 25(OH)D	0.78	0.63–0.97	0.028	0.79	0.63–0.99	0.042
**Colorectal cancer** (n = 102)						
25(OH)D (nmol/l)			0.370			0.672
<50	1.20	0.74–1.94		1.12	0.64–1.84	
50–75	1			1		
>75	0.82	0.52–1.31		0.88	0.55–1.40	
Per 10-nmol/l decrease in 25(OH)D	1.06	0.96–1.16	0.245	1.04	0.94–1.14	0.481
Halving of 25(OH)D	1.29	0.89–1.86	0.178	1.20	0.80–1.79	0.380
**Lung cancer** (n = 93)						
25(OH)D (nmol/l)			0.273			0.338
<50	1.46	0.86–2.48		1.38	0.81–2.34	
50–75	1			1		
>75	1.38	0.86–2.21		1.37	0.85–2.21	
Per 10-nmol/l decrease in 25(OH)D	0.98	0.90–1.08	0.735	0.97	0.88–1.07	0.564
Halving of 25(OH)D	0.97	0.64–1.47	0.886	0.92	0.60–1.41	0.707

Abbreviations: SHR, sub-hazard ratio; 95% CI, 95% confidence interval; 25(OH)D, 25-hydroxyvitamin D.

a Adjusted for age, education, living circumstance, smoking status, physical activity, Charlson Comorbidity Index, body mass index, creatinine, seasonality, and previous diagnosis of cancer (other than the cancer of interest) before blood sampling.

**Table 3:** Competing risks proportional hazards models exploring associations between vitamin D and incident cancers after exclusion of cases diagnosed within 3 years of blood sampling.

	Univariate			Multivariate^a^		
	SHR	95% CI	P value	SHR	95% CI	P value
**Prostate cancer** (n = 155)						
25(OH)D (nmol/l)			0.010			0.027
<50	0.74	0.46–1.19		0.76	0.46–1.23	
50–75	1			1		
>75	1.43	1.02–2.01		1.39	0.98–1.97	
Per 10-nmol/l decrease in 25(OH)D	0.90	0.85–0.95	<0.001	0.91	0.86–0.96	0.001
Halving of 25(OH)D	0.56	0.41–0.77	<0.001	0.58	0.42–0.80	0.001
**Colorectal cancer** (n = 60)						
25(OH)D (nmol/l)			0.722			0.758
<50	1.01	0.53–1.92		1.02	0.53–1.93	
50–75	1			1		
>75	0.80	0.44–1.45		0.80	0.43–1.50	
Per 10-nmol/l decrease in 25(OH)D	1.00	0.90–1.12	0.937	1.00	0.89–1.13	0.946
Halving of 25(OH)D	1.01	0.62–1.65	0.967	1.02	0.60–1.74	0.946
**Lung cancer** (n = 60)						
25(OH)D (nmol/l)			0.663			0.654
<50	1.20	0.61–2.33		1.07	0.54–2.10	
50–75	1			1		
>75	1.30	0.73–2.31		1.31	0.73–2.35	
Per 10-nmol/l decrease in 25(OH)D	0.96	0.86–1.07	0.410	0.93	0.83–1.05	0.227
Halving of 25(OH)D	0.80	0.49–1.32	0.384	0.72	0.42–1.22	0.218

Abbreviations: SHR, sub-hazard ratio; 95% CI, 95% confidence interval; 25(OH)D, 25-hydroxyvitamin D.

a Adjusted for age, education, living circumstance, smoking status, physical activity, Charlson Comorbidity Index, body mass index, creatinine, seasonality, and previous diagnosis of cancer (other than the cancer of interest) before blood sampling.

### Association between 25(OH)D and incident colorectal cancer

In both univariate and multivariate models (Table 2), there was no association between 25(OH)D concentration and incident colorectal cancer. Older age (SHR 1.20, 95% CI 1.11–1.31) and former smoking (SHR 1.68, 95% CI 1.06–2.66) were associated with increased incidence of colorectal cancer in all multivariate models. When cases diagnosed within 3 years of blood sampling were excluded from the models, no association between 25(OH)D and incident colorectal cancer was found (Table 3).

### Association between 25(OH)D and incident lung cancer

In both univariate and multivariate models (Table 2), there was no apparent association between 25(OH)D concentration and incident lung cancer. Older age (SHR 1.12, 95% CI 1.05–1.19), current smoking (SHR 38.9, 95% CI 11.50–131.89) and former smoking (SHR 12.8, 95% CI 4.03–40.67) were associated with increased risks of lung cancer in all multivariate models. When cases diagnosed within 3 years of blood sampling were excluded from the models, no association between 25(OH)D and incident lung cancer was found (Table 3).

**Figure 1:**
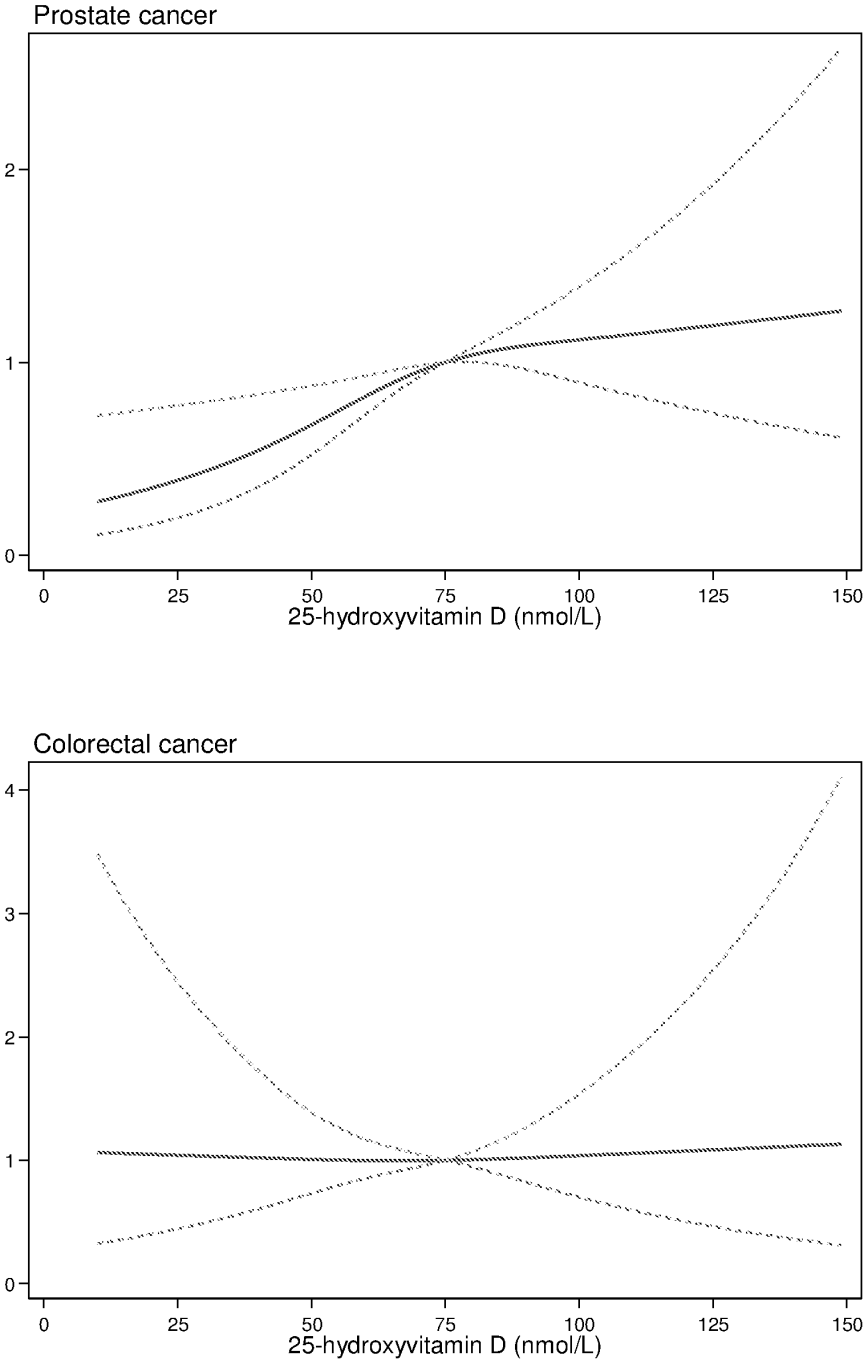
Univariate competing risks proportional hazards models exploring associations between 25-hydroxyvitamin D [25(OH)D] concentrations with incident prostate, colorectal and lung cancers (excluding cancers diagnosed within 3 years of blood sampling). 25(OH)D were entered into the models as restricted cubic splines, with reference value for sub-hazard ratio (sub-HR) of 75 nmol/l. Dashed lines denote 95% confidence intervals.

Figure 1 further illustrates the SHR of incident prostate, colorectal and lung cancer, excluding cancers diagnosed within 3 years of blood sampling, across concentrations of 25(OH)D. Men with 25(OH)D levels <75 nmol/l had a lower SHR for incident prostate cancer, while 25(OH)D levels were not associated with risk of colorectal or lung cancer.

## Discussion

In this prospective cohort study of older men aged 70 years and over, lower 25(OH)D levels were associated with reduced incidence of prostate cancer. Men with plasma 25(OH)D <50 nmol/l had a lower incidence of prostate cancer in comparison to men with 25(OH)D concentrations in the range of 50–75 nmol/l. There were no significant associations between 25(OH)D with incident colorectal and lung cancers in older men.

Our observation that lower 25(OH)D concentrations are associated with reduced risk of prostate cancer is concordant with some of the findings derived from a longitudinal nested case-control study conducted by Tuohimaa et al [17]. This study explored the association between vitamin D and prostate cancer risk (622 prostate cancer cases identified) in Nordic men aged 40–58 years at onset. The authors reported that in middle-aged Norwegian and Swedish men, increased cancer risk was observed for the highest compared to the lowest quintile of 25(OH)D values (i.e. ≥80 versus ≤19 nmol/l; odds ratio [OR] 1.4, 95% CI 0.9–2.1 and OR 1.7, 95% CI 0.7–3.9, respectively). When the Finnish study data was included in analyses, a similar risk pattern persisted (25(OH)D concentration ≥80 nmol/l versus 40–60 nmol/l; OR 1.7, 95% CI 1.1–2.4) [17]. Subsequent findings from the Health Professionals Follow-up Study reportedly showed that men with 25(OHD) levels <37.5 nmol/l had significantly lower risk of poorly differentiated prostate cancers than men with higher levels (OR 0.42, 95% CI 0.23–0.73) [18]. A prospective study by Ahn et al comprising 749 cases of prostate cancer also detected a significant trend of association between increasing quintiles of 25(OH)D levels with increased risk of aggressive disease [19]. Our study indicates that the paradoxical association of lower vitamin D levels with reduced incidence of prostate cancer extends to older men. These findings are contrary to the predominating hypothesis that vitamin D might be beneficial in terms of protecting against cancer risk.

The paradoxical association might be explained by the presence of intraprostatic synthesis of 1,25(OH)D3 from 25(OH)D by normal human prostate cells. This occurs via the expression of 25-hydroxyvitamin D-1a-hydroxylase (1-aOHase) which is diminished in prostate cancer cells [20]. The autocrine synthesis of 1,25(OH)D3 provides a mechanism by which local exposure to increased 25(OH)D could inhibit the growth of prostate cancer. When 1-aOHase expression is diminished in prostate cancer cells, the cancer growth-inhibitory response by 25(OH)D is reduced [21]. Whitlatch et al demonstrated that transfection of the 1-aOHase cDNA into prostate cancer cells with null 1-aOHase expression effectively restores the antiproliferative activity of 25(OH)D in the transfected cells, further supporting the causal association between loss of the enzyme activity with prostate carcinogenesis [22]. Therefore, the effect of local synthesis of 1,25(OH)D3 in the prostate might not be captured in epidemiological studies based on circulating levels of vitamin D. This phenomenon might help to explain the heterogeneous conclusions in other studies exploring the relationship between vitamin D with prostate cancer [23]–[27]. Whilst there is lack of conclusive evidence on the benefit of vitamin D supplementation in the development of prostate cancer, previous studies on the effect of pre-existing prostate cancer have so far produced ambiguous results [28], [29]. A research team in the United States explored the influence of vitamin D3 supplementation at 4000 IU daily for one year on the outcome of early stage, low-risk prostate cancer (Gleason score ≤6, prostate-specific antigen [PSA] ≤10, clinical stage T1c or T2a). More than half of the study subjects remained stable or improved with supplementation, compared to a fifth of the control group who did not receive supplementation (p = 0.025). Conversely, vitamin D3 supplementation did not benefit 40% of the subjects in this open-label clinical trial [28]. Another study involves the randomization of 37 patients with histologically proven adenocarcinoma of the prostate who had selected prostatectomy as primary therapy. Calcitriol was administered to the treatment group at 0.5 µg/kg per week for a 4-week period prior to surgery. When prostatectomy specimens were processed and analyzed, VDR expression was significantly reduced in samples from calcitriol-treated patients (p = 0.004) but there was no statistically significant difference in the fraction of cells expressing the specific molecules involved with cell-cycle regulation and proliferation [29]. With differing model studies and methodologies yielding inconsistent observations, further carefully planned clinical trials of adequate power are warranted to determine whether vitamin D supplementation could alter prostate cancer progression.

In our cohort of older men whose baseline ages ranged between 70 and 88 years, we did not find any significant association between 25(OH)D levels and incident colorectal cancer. The results from previous observational studies have been inconsistent. Enrolled participants in these studies were mostly younger, with the oldest participant being <80 years of age. In a large nested case-control study involving more than 500 000 participants from 10 western European countries (1248 cases of incident colorectal cancer), lower levels of 25(OH)D were associated with higher colorectal cancer risk and higher levels of 25(OH)D associated with lower colorectal cancer risk, in comparison to a pre-defined mid-level concentration of 25(OH)D (50–75 nmol/l). The association was also noted to be stronger in the colon versus the rectum [30]. Results from other studies were also suggestive of a protective effect of vitamin D on colorectal cancer [31], [32]. On the other hand, research by Otani et al [33] and Braun et al [34] did not establish an association. In a 2011 meta-analysis of 9 studies comprising 2767 cases and 3948 controls, an inverse association between 25(OH)D levels and colorectal cancer risk was reported [35]. Several reasons for the discrepancy in findings from these epidemiological studies have been postulated, including residual confounding, the lack of definitive cut-off points for the categories of plasma 25(OH)D levels, and the possibility of publication bias in systematic reviews as small studies with null results might not be accepted for publication. To address these limitations, randomized controlled trials have been conducted, with the largest study involving 36 282 postmenopausal women [36]. Over a seven year period, the incidence of invasive colorectal cancer did not differ between women assigned to calcium plus vitamin D supplementation and those assigned to placebo (168 versus 154 cases; hazard ratio 1.08, 95% CI 0.86–1.34) [36]. Similarly, a smaller study of 2686 participants suggested no benefit of vitamin D treatment [37]. Further research is however needed with more focus on males, and consideration given to increasing the power of future trials, lengthening follow-up, as well as administrating moderate doses of vitamin D in order to generate a clear contrast in 25(OH)D levels between the treatment and control groups.

Prospective cohort studies on the association between 25(OH)D levels and incident lung cancer have also yielded divergent results. Our findings of no significant association are consistent with those derived from a Finnish cohort study of 6937 men and women, from which 122 incident lung cancer cases were identified after a maximum follow-up period of 24 years. After adjustment for age, sex, marital status, educational level, BMI, alcohol consumption, smoking and season of baseline 25(OH)D measurement, the relative risk (RR) for the highest versus lowest tertile of 25(OH)D values was 0.72 (95% CI 0.43–1.19). When the analyses were stratified by gender, 25(OH)D was significantly associated with lung cancer incidence among women (RR 0.16, 95% CI 0.04–0.59) but not among men (RR 1.03, 95% CI 0.59–1.82) [38]. In another case-control study (500 incident lung cancers) involving Finnish male smokers aged 55 and 62 years, no apparent association was observed when using season-specific and season-standardized 25(OH)D measures in the analyses [39]. Similarly, a population-based cohort study conducted by Ordonez-Mena et al in Southwest Germany did not yield any significant association in multivariate models [40]. On the other hand, a sub-analysis in a Danish population of middle-aged men and women has reported a significant risk of lung cancer for a one-unit (2.5 nmol/l) reduction in 25(OH)D concentration (hazard ratio 1.19, 95% CI 1.09–1.31). Whilst the analysis did not show a significant interaction of 25(OH)D with gender on the risk of tobacco-related cancers in this study, interaction on the risk of lung cancer risk specifically was not further explored [41]. Despite inconclusive studies, a role for vitamin D in the development or progression of lung cancer remains plausible as the metabolically active 1,25(OH)D has been demonstrated in animal models to have inhibitory actions on the metastasis and angiogenesis in lung cancer cells [42].

The strengths of our study include the large population-based sample, availability of a wide range of 25(OH)D concentrations to investigate our hypotheses, and adjustment for competing risks in our analyses. Our focus on this well-characterized cohort of older men aged 70 years and above was highly relevant in the study of cancers, with older age being an established risk factor for these adverse health outcomes. However, there were some limitations in this study, including a single blood sample, and the absence of calcium and parathyroid hormone data which might influence vitamin D metabolism. We did not have updated data on physical activity during Wave 2 and therefore cannot dismiss the possibility that this might have altered over time or during the follow-up interval. Our analyses assumed that engagement in physical activity would have been relatively stable between Waves 1 and 2, or at least that its impact would not have substantially altered the outcomes of the study. The interpretation of our findings must take such a caveat into account. There was also limited information on cancer grade or family history of cancer. We were unable to explore the effects of increased PSA testing in our population leading to possible diagnosis of subclinical or low-grade prostate cancer. Prostate cancer diagnoses within our cohort were mostly based on histopathology and we were thus unable to exclude the possibility of false negatives in our data, although this would likely introduce bias towards the null hypothesis. In a recent population-based analysis of PSA screening in Australian men, 66% of overall PSA testing was reportedly conducted in men <65 years of age. One prostate cancer was detected per every 44.5 men who underwent PSA testing (2.2%), and the utilization of PSA tests for detection of prostate cancer decreased with increasing age [43]. It is therefore likely that we have attained a near-complete capture of endpoints (at least for prostate cancer) via electronic record linkage. Finally, the results obtained with regards to colorectal and lung cancers in this cohort of older men may not be easily generalized to women.

In conclusion, our study suggests that higher levels of vitamin D may be associated with increased prostate cancer risk. We found no evidence that vitamin D levels modulate the risk of colorectal or lung cancer in older men. Of note, men with 25(OH)D levels <50 nmol/l had a lower incidence of prostate cancer, yet levels above this threshold are recommended for bone health in older people [44]. Therefore, further carefully designed studies on vitamin D and the incidence of prostate cancers are warranted to determine whether a causal relationship exists.
