# Correction: In Older Men, Lower Plasma 25-Hydroxyvitamin D Is Associated with Reduced Incidence of Prostate, but Not Colorectal or Lung Cancer

**DOI:** 10.1371/journal.pone.0109511

**Published:** 2014-09-26

**Authors:** The PLOS ONE Staff

Figure 1 is missing a third graph regarding lung cancer. The complete version of Figure 1, with all three graphs, can be viewed below.

**Figure 1:**
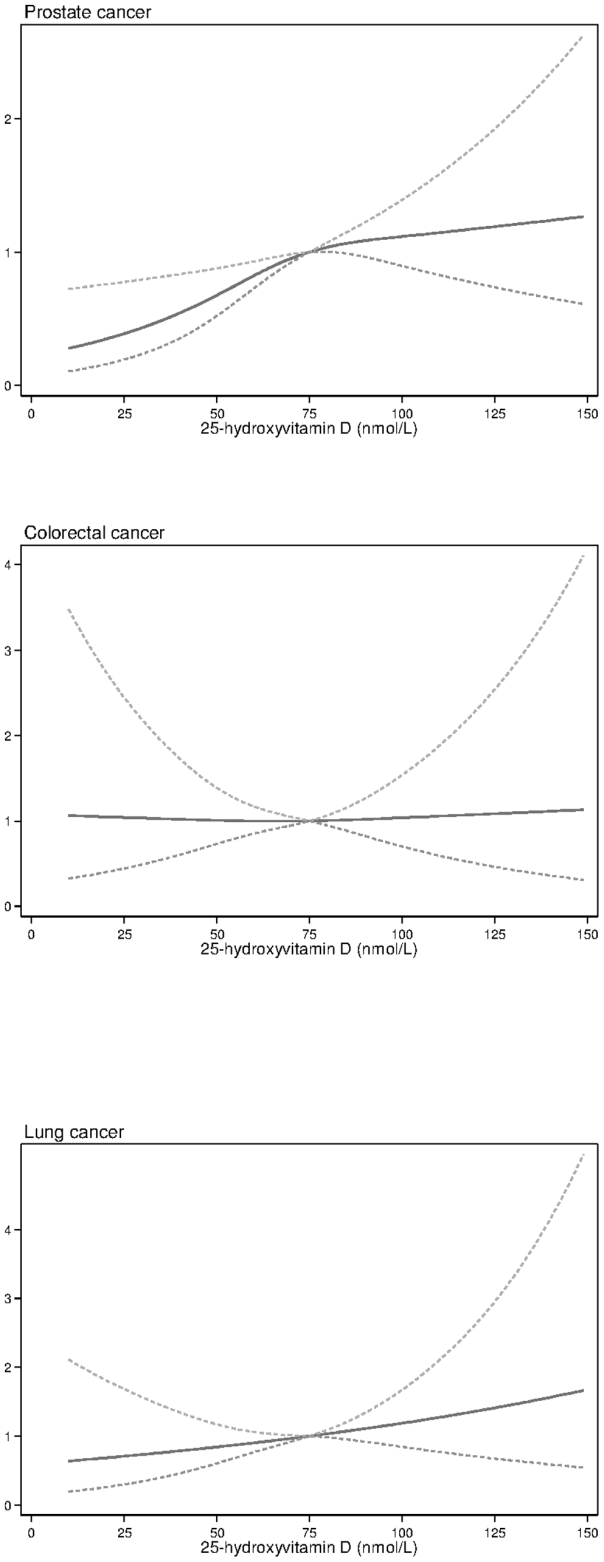
Univariate competing risks proportional hazards models exploring associations between 25-hydroxyvitamin D [25(OH)D] concentrations with incident prostate, colorectal and lung cancers (excluding cancers diagnosed within 3 years of blood sampling). 25(OH)D were entered into the models as restricted cubic splines, with reference value for sub-hazard ratio (sub-HR) of 75 nmol/l. Dashed lines denote 95% confidence intervals.
